# Consequences of Nurse Presenteeism in Switzerland and Portugal and Strategies to Minimize It: A Qualitative Study

**DOI:** 10.3390/healthcare10101871

**Published:** 2022-09-25

**Authors:** Filipa Pereira, Ana Querido, Henk Verloo, Marion Bieri, Carlos Laranjeira

**Affiliations:** 1 School of Health Sciences, HES-SO Valais/Wallis, 1950 Sion, Switzerland; 2 School of Health Sciences, Polytechnic of Leiria, Campus 2, Morro do Lena, Alto do Vieiro, Apartado 4137, 2411-901 Leiria, Portugal; 3 Centre for Innovative Care and Health Technology (ciTechCare), Polytechnic of Leiria, Campus 5, Rua de Santo André-66-68, 2410-541 Leiria, Portugal; 4 Center for Health Technology and Services Research (CINTESIS), NursID, University of Porto, 4200-450 Porto, Portugal; 5 Service of Old Age Psychiatry, Lausanne University Hospital, Route de Cery 60, CH-1008 Prilly, Switzerland; 6 Research in Education and Community Intervention (RECI I&D), Piaget Institute, 3515-776 Viseu, Portugal

**Keywords:** employee health, presenteeism, nursing work, quality of care, patient safety, qualitative research, focus group

## Abstract

Nurses exhibit higher rates of presenteeism than other professionals, with consequences for the quality of care and patient safety. However, nurses’ perceptions of these issues have been poorly explored. This study investigated the perceptions and experiences of frontline nurses and nurse managers in Switzerland and Portugal about the consequences of presenteeism and strategies to minimize it in different healthcare settings. Our qualitative study design used video focus groups involving 55 participants from both countries. Thematic analysis of their transcribed discussions revealed six themes surrounding the consequences of presenteeism: the personal impact on nurses’ health and wellbeing, on their family relationships, and on professional frustration and dissatisfaction; the professional impact on work dynamics; the social impact on the quality of care and patient safety and on society’s impressions of the profession. At the individual, collective, and institutional levels, six strategies were evoked to minimize presenteeism: encouraging professionals’ self-knowledge; creating a positive work atmosphere; facilitating communication channels; developing a positive organizational culture; implementing preventive/curative institutional interventions; identifying and documenting situations linked to presenteeism. Nurses’ perceptions and experiences provided a deeper understanding of their presenteeism and revealed underused pathways toward preventing and minimizing presenteeism via bottom-up approaches.

## 1. Introduction

Healthcare systems face increasing pressure to optimize the quality and safety of the care they deliver. The importance of health in the workplace has grown in recent years, with greater social visibility and more attention from employers [1]. Ensuring safe working environments is a target within United Nations 2030 Sustainable Development Goal 8 [2]. As an emerging concept in healthcare settings, presenteeism is coming under the spotlight for healthcare professionals, especially nurses [3,4]. This neologism describes the opposite of absenteeism, i.e., the phenomenon of employees reporting to work even though they are sick or injured [5].

Recent studies have revealed a high incidence of presenteeism in nursing. For example, 52.6% of American nurses reported that they had struggled to concentrate at work more than once in the preceding 4 weeks [4]. The incidences among registered nurses and assistant nurses in Swedish hospitals were 49% and 47%, respectively [6]. Similarly, 50% of Dutch nurses [7] and 55% of Portuguese nurses reported presenteeism [8]. In China, hospital presenteeism was stated by 94.25% of frontline nurses and 82.1% of nurse managers [9].

Presenteeism has always existed and has even been valued from a managerial standpoint [9,10]. Indeed, the values of loyalty to colleagues and teamwork are especially widespread in the nursing profession, and this tends to maintain the myth of the “super nurse phenomenon” [3]. Nurses frequently fulfil a pivot position to ensure the quality and safety of care and its coordination for patients living in the community or in long-term or acute healthcare facilities. Multitasking and heavy workloads can impact nurses’ wellbeing and lead to physical and mental health problems in the workplace, with potentially serious consequences for staff and their patients, as well as for the productivity of healthcare institutions [11].

Previous studies have reported presenteeism’s wide range of consequences: first, on nurses themselves, from the psychosocial viewpoint, involving work–family conflicts [12] or workplace depression or burnout [13]; second, in terms of their specific professional skills, with decreased mental acuity and communication skills, a decline in the quality of care [14], and lower productivity [15]. Presenteeism’s impact on the quality of nurses’ work and consequently on patient outcomes has been reported as increased numbers of adverse events, including medication errors, patient falls, disease transmission, and iatrogenic infections [14,16,17,18]. Presenteeism may also lead to the omission of fundamental nursing interventions, such as toileting, medication administration, health education, and support with food and fluid intake [19]. A negative impact on wards’ culture of care has also been reported [14].

The COVID-19 pandemic continues to highlight the need to address healthcare staff’s occupational health, especially nurses, whose mental [20,21] and physical health [22] have been severely affected over the last 2 years [23]. Because of healthcare systems’ inability to meet all the care needs of every patient during epidemic episodes, presenteeism came under the spotlight [24].

These consequences and the urgent need to address presenteeism among healthcare staff have led to recommendations including the mobilization of more social resources for their wellbeing and performance [25,26]. However, recommendations are rarely proposed by healthcare staff directly but are rather developed by researchers.

Studies on nurse presenteeism have mainly focused on quantifying its prevalence and consequences [14], and there have been few qualitative explorations of perceptions about presenteeism in specific healthcare settings. However, presenteeism is influenced by institutions’ managerial, structural, social, cultural, and organizational contexts. Indeed, the ecological model developed by McLeroy et al. emphasizes that health promotion should focus not only on intrapersonal behavioral factors but also on the multiple-level factors that influence the specific behavior in question [27]. This model helps to identify opportunities for health promotion by recognizing the individual, professional, and social impact of presenteeism behaviors.

Because presenteeism is also a subjective, multidimensional experience, discovering nurses’ perceptions about it might contribute to a more comprehensive understanding of this worrying phenomenon for healthcare systems. There is a need to examine presenteeism on the basis of the life experiences of nursing staff in different healthcare settings [11].

The present study aimed to explore the cross-cultural aspects of nurses in Portugal and Switzerland’s perceptions, attitudes, and experiences about (a) the consequences of presenteeism, and (b) strategies for minimizing it in different acute care, nursing home, and community healthcare settings. These two countries were chosen because, over the last decade, there has been a significant migration of nurses from Portugal to Switzerland [28]. Although working conditions tend to be more appealing in Switzerland, both countries face a deficit of human resources, physical and psychological strains, and high turnover rates [8,29].

## 2. Materials and Methods

### 2.1. Study Design

This qualitative descriptive study was based on data from a larger international project aiming to address presenteeism and its impact on the quality and safety of care [30]. Presenteeism’s contributing factors were presented in a previous paper [31]. The present study used a qualitative, cross-cultural design to explore nurses’ perceptions, attitudes, and experiences about the consequences of presenteeism and strategies to minimize it in different healthcare settings. We gathered data using audio recordings of the video conference interviews of focus group discussions (FGDs).

### 2.2. Setting and Participants

The research team purposefully selected frontline nurses and nurse managers from different healthcare settings in Portugal and Switzerland. In this study, “frontline nurses” comprised nurses providing care directly to patients, whereas “nurse managers” coordinate activities that contribute to a well-functioning ward, appropriate patient care, and staff support and development [32].

The research team in Switzerland organized four FGDs in the country’s French-speaking region: two in long-term residential care facilities (one FGD with frontline nurses; one with nurse managers) and two in community healthcare settings (one FGD with frontline nurses; one with nurse managers). The research team in Portugal conducted four FGDs in acute care hospital settings of the country’s central region: two with frontline nurses active on acute care wards and two with nurse managers. Each FGD was to be composed of 4–8 participants to encourage the emergence of contrasting opinions [33,34].

Participants were selected using purposive sampling. The lead national investigator contacted, when requested to, the directors of the respective institutions by email or telephone, thus respecting the managerial hierarchical procedures related to the eligible participants. At this point, the study’s rationale was explained to the directors and the eligible participants. Participants’ inclusion criteria were (a) to have been employed in their current healthcare workplace for at least 1 month, and (b) to hold at least a bachelor’s degree in nursing science or equivalent. FGDs were conducted online using video conference interviews because of COVID-19 health restrictions. Consequently, an additional condition for all participants was having an internet connection at the time of the FGD.

### 2.3. Data Collection

Between March and July 2021, a total of 55 participants took part in our eight video-conference FGD interviews, which lasted an average of 75 min. In Portugal, no would-be participants declined their invitation or withdrew. In Switzerland, however, 10 directors refused researchers’ invitations to participate because they estimated that nursing teams had high enough workloads already, they were already committed to other research projects, or presenteeism did not occur in their institution.

Before their FGDs, participants were asked to complete and return by email a short questionnaire including items on age, sex, work function, workplace, working hours/week, and years of experience as a nurse in their specific healthcare setting. It also included an item for participants to rate their health status (ranging from 1 = poor to 5 = very good). The trained moderator conducting the video-conference FGD interview welcomed the participants, fully explained the study’s objective, and informed them that a facilitator would be taking notes and providing a summary of the topics discussed and participants’ involvement at the end.

Similar semi-structured interview guides were used in both countries (one for frontline nurses, one for nurse managers), and discussions were conducted in Portuguese or French, according to the country. Researchers tested the interview guides’ relevance and understandability on four healthcare professionals with similar profiles to participants. Each FGD started with the presentation of a situationally and culturally adapted vignette about presenteeism, published previously with the study’s protocol [30]. After allowing participants time to read the vignette, the discussion opened with the following question: “Based on the vignette, what is presenteeism and how does it impact your work?”. Participants were subsequently asked more specific questions. The interview guide was based on a review of the relevant literature and the authors’ practical knowledge of the research area [30].

Frontline nurses were asked about what presenteeism meant to them and how it influenced their job. If they wished to do so, they were encouraged to share and describe a personal experience involving presenteeism (including decision-making processes and consequences). They were invited to talk about their workplace atmosphere in terms of satisfaction and professional recognition, and to think about how nurse managers could act to prevent presenteeism.

Nurse managers were asked about what presenteeism meant to them, about their opinion of this phenomenon inside their healthcare team, and about its potential causes and consequences in terms of the safety and quality of care. At the end of the FGD, they were also encouraged to think about how they, as managers, could better cope with or prevent presenteeism.

### 2.4. Ethics

This study was approved by the Human Research Ethics Committee of the Canton of Vaud (n° 2021–00071) in Switzerland and the Institutional Review Board of the Polytechnic of Leiria (n° CE/IPLEIRIA/44/2020) in Portugal. Research proceeded according to the principles of the Declaration of Helsinki and its later amendments or comparable ethical standards.

At the beginning of each FGD, the moderator asked participants for their agreement to audio-record the session. They were also informed about the voluntary nature of their participation and the possibility to withdraw at any time without explanation, and they were assured that all the data collected would be handled confidentially, with no individuals being identifiable in quotes or the results. Only the research team had access to the original interview files and transcripts. The participants received no compensation of any kind for their participation.

### 2.5. Data Analysis

Immediately following the FGDs, their audio recordings were transcribed into their respective languages by their moderators and facilitators to produce transcriptions that were as close as possible to the interviewers’ insights into the participants [35].

Thematic content analysis was used [36] to highlight and structure the themes emerging from the data and identify the different perspectives shared by the discussion participants. The research teams in Switzerland and Portugal conducted the first stages of the analysis separately since the transcripts were in their discussion languages. This began by reading and annotating the transcriptions to become familiar with the data. The coding process was then initiated using WebQDA^®^ software to support qualitative data analysis in a collaborative and distributed environment. Initial codes were deduced on the basis of previous research and our conceptual framework. The conceptual framework used the Rainbow and Steege [3] model of presenteeism in nursing and the underlying relationships among presenteeism’s antecedents, definition, and consequences. This was combined with the precipitating factors of presenteeism suggested by Pit and Hansen [37]. Next, the study’s authors repeatedly reviewed the themes and subthemes that appeared across the entire dataset. The authors consulted several times to define and name the themes, and their agreement rate was above 80%. Once each country had established a coding tree, a member of the Swiss research team, of Portuguese mother tongue, met with the Portuguese team to establish a common coding tree inspired by the ecological model of health promotion described by McLeroy et al. [27]; data from both countries were analyzed together, and shared themes and subthemes were developed using English as a common language. All the themes were summarized, and the most significant verbatim quotes were selected to make their synthesis more practical and understandable. The quotes were translated into English and then back-translated into Portuguese and French to ensure their precise meaning had been maintained.

### 2.6. Quality Assurance Methods

The present study followed the guidelines of the Consolidated Criteria for Reporting Qualitative Research (COREQ) [38]. FGDs were conducted according to Krueger and Casey’s methodological guidelines [33].

Furthermore, the four key components of trustworthiness identified by Lincoln and Guba [39]—credibility, dependability, confirmability, and transferability—were used. In both countries, credibility was ensured by conducting a briefing before each FGD, by reviewing the FGD data collected in each interviewer’s written notes on the interview process, and by following each aspect of the interview guidelines. Confirmability was ensured by the team members evaluating the research process during meetings and by reading and analyzing the data together, by describing participants’ demographic data, and by including their direct quotations. Dependability was ensured by clearly defining each stage in the study, by keeping research diaries, by having regular weekly coordination meetings, and by accurately coding data. Strategies to support transferability included a description of participants’ characteristics and the context of their perceptions.

## 3. Results

### 3.1. Participants’ Characteristics

The study’s 55 participants included 49 (89.1%) women and six (10.9%) men (Table 1), with an average age of 45.0 (±8.5) years old. The overall mean self-reported evaluation of health status score was 3.7 out of 5.

### 3.2. Focus Group Findings

**Figure 1:**
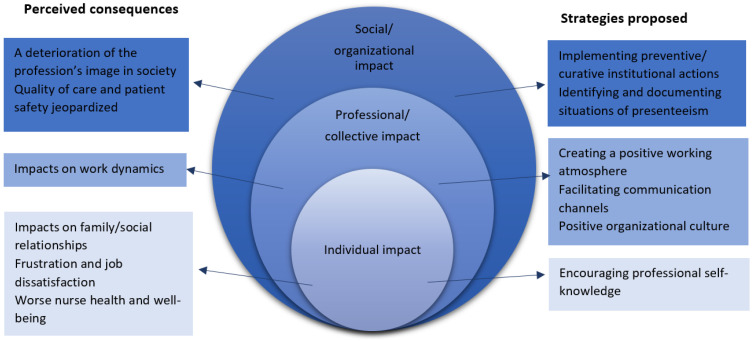
Summary of the findings: consequences of presenteeism and strategies to minimize it inspired by the ecological model of health promotion [27].

The analysis revealed three overarching themes on the consequences of presenteeism and the strategies used to minimize it. These themes were then aligned with the ecological model of health promotion proposed by McLeroy et al. at three levels of analysis (Figure 1) [27].

#### 3.2.1. Consequences of Presenteeism

Three broad themes emerged from the many consequences of presenteeism (Table 2). The first concerned the consequences for nurses themselves, at an individual level, and how they affected their interpersonal relationships. The second broad theme related to the consequences on nursing teams in terms of organization and workload management. The final theme highlighted presenteeism’s consequences on the quality and safety of patient care.

FGD participants revealed that presenteeism had a direct impact on both their physical and their psychological health since it insidiously instituted a form of vicious circle: by refusing to take sick leave when they are ill, nurses frequently resort to self-medication to feel fit enough to come to work and fail to take the time to recover fully. Their strategy is to continue working for as long as possible until continuing becomes untenable.

*“You self-medicate to come to work. I don’t know… a cold. I can’t see myself staying at home because I’ve got a simple little cold: I haven’t got to that state yet… You really wait until the last moment to allow yourself—and I mean the words ‘allow yourself’—to stay at home or to call colleagues and say, ‘Oh, I really can’t make it in right now.’”*

FGD6 (frontline nursing home nurses, Switzerland)

This strategy negatively affects nurses’ health status during and after their presenteeism and an eventual decision to stay at home. Not only do they not fully recover, but this process increases the risk of developing psychological conditions such as depression or burnout.

*“It’s a snowball effect that—if it doesn’t get interrupted—will generate depression and wear down the professional.”*

FGD2 (frontline acute care nurses, Portugal)

A lack of concentration and motivation at work was noted as a consequence of presenteeism. One nurse manager also referred to job dissatisfaction caused by presenteeism (acute care nurse managers, Portugal). A feeling of frustration may emerge, toward the institution and toward themselves, and it is initiated by nurses’ awareness of the workplace mechanisms inciting nurses to go into work. Nurses become conscious of these insidious mechanisms and internalize them, as one nurse described:

*“Actually, I am very aware of how many times I have ended up completely exhausted at the end of these few days, and then I say to myself, ‘Next time, listen to yourself! You shouldn’t have done that!’”*

FGD7 (frontline community healthcare nurses, Switzerland)

Nurses also described presenteeism’s consequences on the social relations within their own families. The distress accumulated during the day does not magically disappear once they return home, and their “relational availability” may be affected.

*“Often, being sick, I come home after work and have to face all the family dynamics… the children… not to mention my husband. It’s extremely difficult (…) and there ends up being conflict. The children don’t realize when you’re out of patience.”*

FGD1 (frontline acute care nurses, Portugal)

The emotional distress felt also leads to a decrease in self-esteem, according to one manager nurse interviewed, which can then impact a nurse’s social sphere when they resort to presenteeism.

*“The way the professional sees themself changes; they feels that they are no longer useful in their care unit, that they cannot give more (…) This will also have consequences socially. We are talking about consequences in social interactions, with increasing isolation.”*

FGD4 (acute care nurse managers, Portugal)

The potential consequences inside nursing teams, in terms of organization and workload management, were noted previously. Productivity falls when there is presenteeism, and this gets noticed by presentee nurses and the rest of the nursing team.

*“(…) on shift changes, I’m sitting looking at my colleagues and thinking ‘These people are not capable of doing everything that is required of them’. A professional loses motivation and is no longer as productive as they should be.”*

FGD1 (frontline acute care nurses, Portugal)

In a chain reaction, presentee nurses’ lack of productivity can increase workloads for the rest of their team. However, making a categorical distinction between these two types of actors does not reflect the workplace reality since a “contagion” phenomenon can develop within the healthcare team, with each actor gradually finding themselves having to resort to presenteeism.

*“It is a professional phenomenon that somehow ends up spreading to the whole team, being almost contagious… it’s that phenomenon of the ‘bad apple’ in the fruit basket that ends up infecting the whole team, because when things don’t get done or mistakes are made, gaps have to be plugged, and sometimes from shift to shift.”*

FGD1 (frontline acute care nurses, Portugal)

Relational consequences within teams were identified, such as failing solidarity, less collaborative spirit between nurses, and a growing spirit of individualism. Presentee nurses may even become “aggressive” and “irritable” with team members or patients, creating a challenging and problematic workplace atmosphere.

*“She became irritable. She was so afraid of missing something herself that she was always repeating instructions to the team. She became a little bit aggressive despite herself. And then, I had to call her into a meeting to precisely reframe things, to set objectives.”*

FGD5 (nursing home nurse managers, Switzerland)

The negative impact on the relational climate within a team and toward patients may go hand in hand with a negative impact on the quality of care. When a presentee nurse no longer has the availability to listen empathetically or attend to suffering patients, or when the rest of the team’s workload seems to have multiplied manyfold, care may become severely depersonalized:

*“(…) results in a bad relationship with patients and depersonalization of care. We do everything in a rush because we don’t have the mental availability to be there listening to them [patients].”*

FGD2 (frontline acute care nurses, Portugal)

The proper standards of the quality of care may, therefore, no longer be guaranteed, and the risk of committing errors, particularly concerning medication, increases, as testified to by a nurse who had once found herself in this situation.

*“I can’t say that I had a real illness. There wasn’t a diagnosis, but I was hugely fatigued, and I was unwell. And, well, I made a medication error. And if I analyze the situation now, it was because I wasn’t in my right mind: I wasn’t well enough to go to work that day.”*

FGD6 (frontline nursing home nurses, Switzerland)

Lastly, all these potential consequences of presenteeism may contribute to a negative image of the nursing profession:

*“We, as a profession, well, the way we work and the message we send is not the right one. Our image ends up being compromised by our mistakes, by our way of being. After all, we are a class [of professionals]. And that interferes with the view that the people we care for have about us.”*

FGD2 (frontline acute care nurse, Portugal)

Caught up in complex mechanisms and issues that are sometimes beyond their control, presentee nurses may tend to lose sight of their profession’s core mission, which is providing patients with the best possible care.

#### 3.2.2. Strategies to Minimize Presenteeism

Faced with the potential consequences of presenteeism, described above, numerous preventive strategies emerged from the FGD. These strategies involved (1) individuals observing and asserting themselves, (2) intra-team communication and workplace atmosphere, and (3) more generally, structural and organizational issues concerning work settings as a whole (Table 3).

The FGD suggested that, at the individual level, nurses should first be encouraged to be more introspective about their relationships, work–life balance, and health status, and that they should listen to themselves more. One nurse explained:

*“I have headaches. There are several types of headaches. They go from the little headache where you take a Dafalgan; you know that it will pass, so you come to work. You take what you need, and you feel that it will pass. If you feel like it’s not going to pass, you don’t come to work. And with no guilt.”*

FGD6 (frontline nursing home nurses, Switzerland)

Nurse managers evoked the leadership style they might adopt to prevent presenteeism or its consequences. Regarding “setting a good example”, they too should not come to work when in poor health. Once awareness of presenteeism has been raised, FGD participants felt that it was essential to be able to assert themselves with team leaders when the situation required it. One nurse summarized this process of employee empowerment and responsibility:

*“We must know our rights so that we can protect ourselves (…) whenever we have problems, we must negotiate solutions; I think that we can take care of our mental health individually (…) we have to regulate ourselves in order to prevent wear and tear.”*

FGD2 (frontline acute care nurses, Portugal)

Strategies aimed at fostering team spirit and solidarity were proposed to prevent presenteeism. Taking care of each other and being attentive to colleagues’ wellbeing were mentioned as components of a positive, empathic working atmosphere.

*“We took care of that colleague because they were not able to work, and we asked them to go home. It’s about valuing people: observing, giving positive reinforcement, and being able to work with the team.”*

FGD1 (frontline acute care nurses, Portugal)

Participants mentioned the emerging idea that by promoting teamwork and collaboration, the health of the team as a whole could be enhanced.

*“(…) teams are living beings that change and shape themselves as their individual elements grow.”*

FGD3 (acute care nurse managers, Portugal)

According to the nurse participants, creating links between workers and spaces for sharing can promote nurses’ mental health. Using presenteeism as a name for a known phenomenon and having the opportunity to talk about it were ways of valuing peer support alongside other “teambuilding” activities that might take place outside the professional space.

Interviewees believed that improving communication was also a strategy to combat presenteeism. In the spirit of top-down management, nurse managers should get to know their frontline nurses better and show a greater capacity to listen to them and understand any potential workplace suffering. Regular meetings with agendas that went beyond organizational issues would help to promote a closeness to staff.

It would also be managers’ responsibility to remind frontline nurses that—just like any other employee—they have a right to be ill, thus fostering a form of tolerance on this subject:

*“We must be attentive, and our role is to be attentive to each professional’s wellbeing and to be able to reassure a person, to explain to them that they have the right to be sick, that they can be ill, that every effort will be made to replace them without overloading their colleagues, although perhaps it will be someone from outside.”*

FGD5 (nursing home nurse managers, Switzerland)

Nurses also felt that they had a role to play in improving communication, e.g., by taking part in information sessions or conferences. In this way, they could communicate about the consequences of presenteeism and share scientific information about it.

*“Focus on the different communication channels about that. It can be quite simple: an article that you see on burnout and its consequences, on exhaustion, etc., and then, in fact, conveying that information. That’s what I was saying in relation to these meeting spaces—that we can already be aware of this phenomenon through information, by disseminating information.”*

FGD7 (frontline community healthcare in Switzerland)

Thus, focusing on better communication tends to create a positive organizational culture in itself, on the one hand, by promoting a leadership style focused on employee wellbeing and, on the other, by encouraging active employee participation.

*“(…) involving people in defining the team’s goals, even institutional goals, makes everybody responsible for the issue. And, therefore, I think that this co-accountability can translate into a lower level of presenteeism.”*

FGD1 (frontline acute care nurses, Portugal)

Participants also mentioned institutional- and organizational-level elements that could be put in place to reduce presenteeism. Preventive measures, such as the mandatory recovery of overtime or the implementation of a pool nursing service, could play a major role in nurses’ decisions on whether or not to come to work when they feel ill.

Adapting working environments for nurses with particular needs is also a way to prevent them from resorting to presenteeism.

*“But it’s true that presenteeism means that sometimes, because they absolutely want to keep on working, you have to find ways for them to work and be safe too. So this can quickly require some difficult adaptation.”*

FGD8 (community healthcare nurse managers, Switzerland)

Healthcare institutions should also support the promotion of nurses’ mental health. FGDs revealed that using stress-reduction workshops (e.g., brief sessions of yoga or relaxation) at the beginning of the day could be preventive measures to raise awareness of the central role of mental health in the workplace, thus preventing presenteeism. Interviewees also mentioned the organization of supervisory programs for nurse managers aimed at providing them with tools focusing on emotional support. When a nurse resorts to presenteeism, collaboration with other departments or specialists within the institution is key, according to the interviewees; referring the presentee to a psychologist or an occupational physician, or discussing the case with the human resources department, can be important tools for nurse managers. It is also essential that clear procedures and steps for dealing with presenteeism are set out, as is already often the case for absenteeism.

*“(…) For someone who regularly resorts to absenteeism—to see the person and all—we have a whole procedure to go through and which is written down, but (…) there’s nothing written down in relation to presenteeism. There is no procedure. It’s really about taking the initiative to meet with them, but we don’t have a written procedure. Indeed, this is something that could be put in place.”*

FGD5 (nursing home nurse managers, Switzerland)

The nurses interviewed suggested that these procedures could include documenting each case of presenteeism in order to gain an in-depth understanding of the presentee’s situation and prevent such difficulties from recurring, including for other nurses. This last measure would give visibility and “legitimacy” to presenteeism since there would be steps to follow and a procedure to adopt when it was observed. With this in mind, quantifying and explaining the costs of presenteeism are essential steps toward raising collective awareness of the phenomenon and developing preventive measures.

*“(…) we are really living in an age where everything has to be quantified, weighed, and measured, and if we don’t have that, I have a bit of an impression that we are talking into the void. Because we can talk, but things are not moving forward. Because we must not only talk about the frequency of the problem, but above all what it costs. Because that’s the big argument. Because as long as it costs nothing, who cares. But if we finally start to quantify everything that presenteeism costs an institution, well then, maybe mentalities might change.”*

FGD7 (frontline community healthcare nurses, Switzerland)

## 4. Discussion

This international multicenter study revealed the perceptions of frontline nurses and nurse managers in Switzerland and Portugal with regard to presenteeism, its consequences, strategies to minimize it, and the roles of specific healthcare settings in forming those perceptions.

Research was conducted in these two countries to more broadly map opinions from different sociocultural contexts, even though nurses in both countries face similar work-related issues: a lack of human resources, physical and psychological stressors, job insecurity, and high staff turnover rates [8,29]. Our study noted cultural differences in the participant recruitment phase; in contrast to the research team members in Portugal, the Swiss research team received numerous refusals to participate. Nevertheless, these refusals came from directors and/or nurse managers and not from nurses themselves. This could be explained by the fact it is usually more appropriate for research projects in Switzerland to work from the top of an institutional hierarchy (the institution’s human resources manager or managing director) or to ask a director’s or nurse manager’s permission for staff to participate. In contrast, in Portugal, contacting healthcare professionals directly is quite acceptable. Thus, nurses in Switzerland did not have the same opportunities to participate as in Portugal. Nevertheless, the reasons for refusal given by directors or nurse managers in Switzerland were a relevant finding for research on the phenomenon of presenteeism. Previous studies have found that nursing workloads hinder their participation in new studies, as does the belief that the phenomenon of presenteeism does not exist in their institution [40].

Participants’ work rates were different. The nurses in the recruiting hospital in Portugal all worked full-time, at 100%, whereas in Switzerland, nurses often chose to work part-time, at lower rates [41]. As shown in quantitative design studies [29,40], the number of working hours did not affect perceptions of presenteeism, contrary to what one might have assumed (i.e., that nurses with lower work rates resort less frequently to presenteeism). Indeed, our results suggested that even nurses who did not work full time might resort to presenteeism.

Although cultural differences between the two countries were notable in the recruitment strategy and work rate, perceptions of the consequences of presenteeism and the strategies to minimize it were aligned in both countries. Although Webster and Liu [40] revealed higher levels of presenteeism among nurses working in hospitals than in long-term residential care, our findings showed no differences between nurses’ perceptions of the consequences of presenteeism in acute care, nursing home, or community health settings.

In contrast, different perspectives emerged depending on the hierarchical position held. Frontline nurses and nurse managers presented fairly different perceptions of the consequences of presenteeism and the remediation strategies proposed. Whereas frontline nurses spoke about their own experiences, nurse managers generally spoke about the perceived presenteeism in their nursing teams.

Regarding perceptions of presenteeism’s consequences, our results reinforce previous findings in the literature but also provide new information. Our participants corroborated several studies revealing that presenteeism affects the quality of nursing and patient care outcomes [14,16,42]. Although fewer studies have explored the impact of presenteeism on nurses themselves, our findings complemented those of Brandford and Reed [43] and Jun and Ojemeni [13] regarding workplace depression and burnout, and those of Camerino and Sandri [12] regarding the impact of work schedules on work–family conflicts among Italian nurses.

To the best of our knowledge, presenteeism’s impact on nursing teams has been explored less often [14]. However, our findings showed the importance of considering it, not only because of the risks of interpersonal conflicts with peers and patients but also because of the peer “contagion” effect. Presenteeism by one team member overburdens the other members, worsening their working conditions and eventually leading to them resorting to presenteeism as well. Thus, presenteeism’s consequences seem to be linked to a vicious circle where presenteeism can be considered a consequence of different risk factors and a precipitating factor; a presentee nurse’s lack of productivity increases workloads for the rest of their team as a whole (consequence), but the increased workloads also contribute to presenteeism (precipitating factor). This is a call to managers to develop employee-centered resource policies that can prevent the phenomenon of presenteeism from worsening and spreading within the team.

Frontline nurses and nurse managers from both countries proposed various potential strategies for minimizing presenteeism using pragmatic, co-constructed approaches that could be embedded in daily practice. Most of the proposed strategies entailed team-level collective and collaborative actions. These would have to be implemented in a spirit of systemic flexibility, adapting to the changes that the team may encounter. Participants also suggested organizational- and structural-level strategies. Healthcare managers should use tangible actions to create more reasonable regulations and humane, employee-centered management systems for nurses to reduce presenteeism, financial costs, or falls in the quality and safety of care. This supported findings by Shan and Wang [9] in their quantitative cross-sectional study about the prevalence, consequences, and causes of presenteeism among Chinese nurses.

Nursing and healthcare managers should abandon any models focused solely on absenteeism and familiarize themselves with the concept of presenteeism and its harmful consequences. Implementing strategies aimed at reducing or preventing presenteeism could lead not only to lower levels of presenteeism and absenteeism but also to healthier, more productive nurses with higher levels of job satisfaction and greater safety for all hospital users [5].

In addition, nurses themselves have the potential to make use of their experiences of presenteeism by being more assertive in the workplace and promoting a better daily work–life balance. As suggested by van den Heuvel and Demerouti [44], nurses should self-regulate their positive attitudes to change by using meaning-making to reflect on how change might support their goals and values, all of which positively relate to their commitment to their work and to their profession.

Although many recent studies focused on overburdened nurses during the pandemic crisis [45], all the same circumstances were mentioned during our FGD interviews, but not systematically. This suggests that, although the phenomena of heavy nursing workloads and presenteeism were particularly highlighted during the COVID-19 pandemic [45,46], they were far from being new concerns for the profession.

### 4.1. Study Strengths and Limitations

The originality of our research consisted of questioning—using a qualitative methodology involving an FGD and a vignette—frontline nurses and nurse managers about their perceptions of presenteeism’s consequences and the strategies that they believed could counteract it. To the best of our knowledge, this was a new and original approach, unmentioned in the literature, with which to collect and analyze data on presenteeism. FGDs have several advantages, notably organizational ones, as they allow different views on the same subject to be collected from multiple actors at the same time [47]. From the participant’s point of view, FGDs provide a less formal atmosphere in which to share their point of view and interact than does an individual interview; since their points of view could be shared by others in the group, they may feel more comfortable expressing themselves [47]. However, each moderator was aware of biases affecting FGDs, such as the dominance effect and the halo effect [47].

In addition, an introduction to the topic was made using a vignette, which is a useful tool with which to explore professionals’ perceptions of complex work tasks such as decision-making and to address the context-specific conditions of daily professional activities [48,49]. With regard to the participants’ profiles, their heterogeneous ages, years of experience, professional training, and sex helped a variety of experiences to emerge.

This international multicenter study allows us to suggest that our findings might be transferable to similar healthcare settings in the participating countries, although some precautions of interpretation should be taken into account.

Data were collected using audio-recorded online FGDs, considered to be characterized by synchronous communication in time but asynchronous communication in place [50]. Because using face-to-face FGDs could have been compromised by the ongoing pandemic situation, the research team decided to conduct them online [51,52]. This may have prevented the participation of some nurses who may not have felt confident with the technical requirements for online interviews or who may not have felt confident about online confidentiality [50]. However, online technologies are increasingly used in qualitative data collection in general [53] and have demonstrated their ability to enable continuity in research processes within the context of the COVID-19 pandemic [50,51].

Given the difficulties of participant recruitment in Switzerland, the FGDs there were conducted with just four participants each instead of the 6–8 initially planned. However, this did not seem to affect our results as perceptions of the consequences of presenteeism and the strategies needed to minimize it were congruent in both countries. From a methodological point of view, an FGD can still be conducted with four participants [33].

Cultural organizational differences between healthcare settings (acute, primary, and long-term care) could have impacted our findings; however, these differences were not separately explored for reasons of confidentiality and sampling.

Lastly, our study may have been influenced by Swiss and Portuguese social security systems or labor laws. Indeed, whether salaries are covered in cases of employee illness and how the quality of working conditions is guaranteed could have been determinants in how participants perceived presenteeism, as could human resources strategies to address both absenteeism and presenteeism. However, as discussed above, perceptions of the consequences of presenteeism and the strategies needed to minimize it were aligned in both countries.

### 4.2. Individual, Professional, and Organizational Implications

This study investigated perceptions about presenteeism from the viewpoints of frontline nurses and nurse managers, but it did not investigate nurse managers’ presenteeism. As suggested by Ruhle and Breitsohl [54], future research about presenteeism should also examine the consequences of how managers’ behavior may lead to presenteeism, as well as exchanges between managers and frontline nurses concerning this topic. In addition, future research should explore the mechanisms of interaction between the precipitating factors and consequences of presenteeism. A clear understanding of these underlying mechanisms of interaction would enable institutions and policymakers to develop robust, scientific strategies to help prevent and reduce nurse presenteeism.

Although we broadly categorized perceptions of presenteeism into individual, collective, and institutional consequences and strategies for dealing with it, based on the ecological model of health promotion, these three levels must be considered closely interconnected. The consequences of presenteeism felt at one level could have repercussions on the other two, cyclically aggravating the phenomenon for the nurse, but also those around them, such as family, colleagues, patients, the institution (their employer), and the healthcare system as a whole. For this reason, it seems important to simultaneously implement different strategies at different levels. Indeed, the ecological model of health promotion focuses on making both individual and social environmental factors the targets of health promotion interventions [27]. The model addresses the importance of interventions directed at changing interpersonal, organizational, community, and public-policy factors that might otherwise induce and maintain resorting to presenteeism. The model assumes that appropriate changes in the social environment will produce changes in individuals and that supporting individuals in the population is essential for implementing changes in working environments.

## 5. Conclusions

The present study emphasized several consequences of nurses resorting to presenteeism. These suggest that healthcare administration units and policymakers should pay more attention to nurse presenteeism and take active measures to prevent it. The various strategies suggested during the FGDs highlighted the need to act at several different levels at once: encouraging certain behaviors among nurses themselves, valuing and promoting teamwork, promoting the essential role of nurse managers, and, at the institutional level, enhancing awareness about presenteeism and the legitimacy of taking sick leave. Effective interventions could contribute to promoting nurses’ health and improving the quality of nursing care.

## Figures and Tables

**Table 1:** Participants’ sociodemographic and professional characteristics.

Sociodemographic and Professional Characteristics	Participants (*n* = 55)
Sex	
Female (%)	49 (89.1)
Male (%)	6 (10.9)
Age	
Mean ± SD (range)	45.0 ± 8.5 (25–61)
Country	
Portugal	39 (70.9)
Switzerland	16 (29.1)
Workplace function	
Frontline nurses	28 (50.9)
Nurse managers	27 (49.1)
Years of experience in healthcare	
Mean ± SD (range)	19.3 ± 10.0 (3–40)
Healthcare setting	
Acute care (Portugal) (%)	39 (70.9)
Primary care (Switzerland) (%)	6 (10.9)
Long-term care (Switzerland) (%)	10 (18.2)
Self-reported evaluation of health status (1 = bad; 2 = reasonable; 3 = good; 4 = very good; 5 = excellent)	
Mean (range)	3.7 (2–5)

**Table 2:** The consequences of presenteeism evoked by frontline nurses and nurse managers in both countries.

Broad Themes	Themes	Subthemes
Individual impact (nurse level)	Nurses’ health and wellbeing	The physical, psychological, and mental impact
		Self-medication to keep working
	Frustration and job dissatisfaction	
	Family/social relationships	Failing capacity to manage relationships
		Committed family dynamics
Collective impact (professional level)	Workplace dynamics	Decrease in productivity
		Team overload
		Peer “contagion” effect
		Interpersonal conflict with peers and patients
Social impact (patient and population level)	Quality of care and patient safety	Depersonalization of careCompromised safety of care
	Professional image in society	

**Table 3:** Strategies to minimize presenteeism described by frontline nurses and nurse managers in both countries.

Broad Themes	Themes	Subthemes
**Individual strategies** (**nurse level**)	Favoring professionals’ self-knowledge	Using assertiveness at work
		Promoting a work–life balance
**Collective strategies** (**team level**)	Creating a positive working atmosphere	Valuing teamwork
		Supporting peers (through digital social networks, events, etc.)
	Facilitating communication channels	Supporting communication from the “bosses” to staff
		Being attentive to employees’ needs
		Sharing information coming from employees
	Developing a positive organizational culture	Encouraging collaborative and active employee participation
		Encouraging employee-centered leadership styles
**Institutional strategies** (**organizational and structural level**)	Implementing preventive/curative institutional actions	Planning and adapting working contexts
		Promoting occupational mental health programs
		Preparing procedures for situations of presenteeism
		Ensuring specialized support (from occupational health and psychology professionals)
	Identifying and documenting situations involving presenteeism	Assessing and quantifying the impact of presenteeism

## Data Availability

The data presented in this study are available on request from the corresponding author. The data are not publicly available due to containing information that could compromise the privacy of research participants.
